# Nanohydrogels Based on Self-Assembly of Cationic Pullulan and Anionic Dextran Derivatives for Efficient Delivery of Piroxicam

**DOI:** 10.3390/pharmaceutics11120622

**Published:** 2019-11-21

**Authors:** Dorota Lachowicz, Przemyslaw Mielczarek, Roma Wirecka, Katarzyna Berent, Anna Karewicz, Michał Szuwarzyński, Szczepan Zapotoczny

**Affiliations:** 1 Academic Centre for Materials and Nanotechnology, AGH University of Science and Technology, al. A. Mickiewicza 30, 30-059 Krakow, Poland; roma.wirecka@fis.agh.edu.pl (R.W.); kberent@agh.edu.pl (K.B.); michal.szuwarzynski@agh.edu.pl (M.S.); 2 Department of Biochemistry and Neurobiology, Faculty of Materials Science and Ceramics, AGH University of Science and Technology, al. A. Mickiewicza 30, 30-059 Krakow, Poland; przemyslaw.mielczarek@agh.edu.pl; 3 Maj Institute of Pharmacology, Polish Academy of Sciences, Smetna 12, 31-343 Krakow, Poland; 4 Faculty of Physics and Applied Computer Science, AGH University of Science and Technology, al. A. Mickiewicza 30, 30-059 Krakow, Poland; 5 Faculty of Chemistry, Jagiellonian University, Gronostajowa 2, 30-387 Krakow, Poland; karewicz@chemia.uj.edu.pl (A.K.); zapotocz@chemia.uj.edu.pl (S.Z.)

**Keywords:** nanohydrogel, self-organization, piroxicam, cationic pullulan, anionic dextran, polyelectrolyte

## Abstract

A cationic derivative of pullulan was obtained by grafting reaction and used together with dextran sulfate to form polysaccharide-based nanohydrogel cross-linked via electrostatic interactions between polyions. Due to the polycation-polyanion interactions nanohydrogel particles were formed instantly and spontaneously in water. The nanoparticles were colloidally stable and their size and surface charge could be controlled by the polycation/polyanion ratio. The morphology of the obtained particles was visualized by scanning electron microscopy (SEM), transmission electron microscopy (TEM) and atomic force microscopy (AFM). The resulting structures were spherical, with hydrodynamic diameters in the range of 100–150 nm. The binding constant (*K_a_*) of a model drug, piroxicam, to the cationic pullulan (C-PUL) was determined by spectrophotometric measurements. The value of *K_a_* was calculated according to the Benesi—Hildebrand equation to be (3.6 ± 0.2) × 10^3^ M^−1^. After binding to cationic pullulan, piroxicam was effectively entrapped inside the nanohydrogel particles and released in a controlled way. The obtained system was efficiently taken up by cells and was shown to be biocompatible.

## 1. Introduction

There are many biologically active compounds which have a potential as highly effective drugs, but their low bioavailability (due to low solubility in aqueous media or instability in physiological conditions) or high toxicity prevent their application. There is, therefore, a high demand for suitable, dedicated carriers. Polysaccharides have been proposed in many biomedical applications [1,2,3,4] Polysaccharide nanohydrogel formulations are recently gaining interest as drug delivery systems, mainly due to their ability to modify the pharmacodynamics and pharmacokinetics of the transported therapeutic agent [1,2,3,4]. The polymeric matrix of the nanogels can entrap significant amount of the bioactive compound (e.g., low molecular weight drugs, proteins, DNA or RNA) and release it in a controlled way [5,6,7,8]. In order to adjust the release profile of a bioactive substance to the needs of the therapy the ability of nanogels to respond to the microenvironmental stimuli such as light, magnetic field, changes in ionic strength, pH, temperature, or redox potential, are often exploited [9,10,11,12,13]. For example, sodium alginate was combined with N-isopropylacrylamide to obtain a dual-responsive system for oxytetracycline delivery, where the drug release could be effectively controlled by both pH and temperature [14]. Chitosan based nanogel which showed pH-dependent drug release which mimicked the skin cancer micro-environment was very recently proposed by Sahu et al. [15].

It is, however, worth noticing, that many of the studied nanogel systems are based on the combination of polysaccharide and synthetic polymer, which often makes them less biocompatible. Other large group of nanogels is made by the hydrophobic modifications of polysaccharides. For these polymers the formation of the stable nanogel usually requires additional steps (solvent removal, heating, ionotrpic gelation, cross-linking).

Pullulan is a natural polysaccharide produced from starch by the fungus *Aureobasidium pullulans*. This water-soluble polymer has a linear structure consisting of repeating maltotriose units [16]. Due to its ordered structure pullulan has film-forming ability, considerable mechanical strengths and adhesiveness, thus it is widely used as a component of composite polymer materials, in the construction of electrospun fibres and gels [16,17,18]. The latter are usually obtained by chemical cross-linking [19], a highly universal method which allows to obtain hydrogels and nanohydrogels with high mechanical stability. However, many of the chemical agents used as cross-linkers are toxic. Furthermore, the cross-linking process may lead to undesirable reactions with active compound encapsulated within a nanohydrogel system [1,20,21]. To avoid these negative effects, physical cross-linking can be used. Ionically cross-linked nanogels can be obtained by microemulsification, liposome templating, or solvent displacement [22,23,24,25,26]. Unfortunately, the presence of small ions in the nanogels obtained in this way significantly affects the transported active substance and its pharmacokinetics. Electrostatic interactions between the positively and negatively charged polymers are becoming an interesting alternative in obtaining nanogel systems [7,27]. Recently, nanocarriers and nanogels prepared by ionic crosslinking of polyelectrolytes [27,28] have been studied in a wide range of biomedical applications, including drug delivery systems [29,30,31,32,33], as well as systems for medical imaging [34,35] and theranostics.

Here we propose a simple drug delivery system based on two polyions derived from naturally occurring, highly biocompatible polysaccharides, pullulan and dextran, which instantly and spontaneously self-assembly to form nanohydrogel particles of adjustable size and surface charge. Due to the electrostatic interactions between two strong polyions, the obtained system should possess high colloidal stability while its size and charge can be optimized to facilitate cell penetration and effective delivery.

For this purpose pullulan (PUL) was first cationically modified by grafting with *N*-acrylamidopropyl-*N*,*N*,*N*-trimethylammonium chloride (APTMAC). Cationic pullulan (C-PUL) was then employed as a building block to electrostatically assembly and crosslink with anionic dextran (DEX-A) in water. As a result a nanogel was formed (C-PUL/DEX-A NG). To test the ability of the obtained system, a model drug, piroxicam, was incorporated into the nanogel particles. Piroxicam, while showing strong analgesic, anti-inflammatory, and antipyretic activity [36,37,38], is only sparingly soluble in water (solubility 2.2 × 10^−5^ M) [39,40]. Thus our novel delivery nanogel was verified as a suitable carrier to facilitate its delivery.

## 2. Materials and Methods

### 2.1. Materials

Piroxicam(4-Hydroxy-2-methyl-3-(pyrid-2-yl-carbamoyl)-2*H*-1,2-benzothiazine 1,1-dioxide, meets USP testing specifications), pullulan, PUL (from Aureobasidium pullulans, *M_w_* 360–480 kDa), dextran sulfate sodium salt (*M_w_* 7–20 kDa, sulfur content (S/C-analysis) 17.0–19.0%), (3-Acrylamidopropyl)trimethylammonium chloride solution–APTMAC (75 wt% in H_2_O), benzoyl peroxide (BPO) and *N*,*N*-dimethylformamide (DMF) were purchased from Sigma-Aldrich. Spectroscopic grade methanol and oleic acid (p.a.) were purchased from POCH, Gliwice, Poland.

### 2.2. Synthesis of Cationic Pullulan (C-PUL)

In a two-necked flask 0.75 g of pullulan was dissolved in 60 mL of DMF. The solution was vigorously stirred with a magnetic stirrer and simultaneously bubbled with gaseous nitrogen for 30 min at 45 °C. Then the solution of BPO (675 mg dissolved in 4.5 mL of degassed DMF) was added. After 10 min a portion of 2.205 g APTMAC (75%, dissolved in 10.5 mL of degassed DMF) was added. The reaction mixture was then heated at 70 °C for 3 h under constant mixing and under constant flow of gaseous nitrogen. Then the mixture was cooled down and dialyzed against DMF for the first 2 days and against water for the following 14 days (dialysis tube MWCO, 14 kDa cut-off). The purified product, C-PUL, was isolated by freeze-drying.

### 2.3. Preparation of C-PUL/DEX-A Nanohydrogel Particles (NGs)

1 mg/mL of C-PUL solution in water and 1mg/mL of DEX-A solution in water were prepared. C-PUL solution was added dropwise to DEX-A solution under vigorous stirring until the desired volume ratio was reached. Nanohydrogels (NGs) were formed instantaneously, which could be observed as an increased turbidity of the mixture. The suspension was stirred for 30 min and then nanogel were separated by centrifugation at 8000 rpm for 20 min at 4 °C and washed four times by re-suspending in distilled water followed by centrifugation and removal of the supernatant. Finally, the nanohydrogel was re-suspended in 2 mL of distilled water and used in further studies.

### 2.4. Preparation of the Piroxicam Loaded Nanogel NG-PIXs

50 μL of 1 mg/mL solution of piroxicam in methanol was added slowly to 1 mL of 1mg/mL solution of C-PUL in water. The resulting solution was then stirred in the water bath at 25 °C for 20 min. During incubation, the solution was also purged with gaseous nitrogen in order to remove methanol. 1 mg/mL DEX-A solution was then added to C-PUL solution containing piroxicam under vigorous stirring until the volume ratio of 1:4 was reached. NGs were formed instantaneously. The suspension was stirred for 30 min and the nanohydrogel was separated by centrifugation at 8000 rpm for 20 min at 4 °C. Purification was done according to the procedure described for piroxicam-free NGs (Section 2.3).

### 2.5. Characterization of Pullulan Derivative

FT-IR spectrum was recorded on Bruker IFS 48 (Bruker, Ettlingen, Germany) using Thermo Scientific iD5 Diamond ATR accessory (Thermo Fisher Scientific, Waltham, MA, USA). ^1^H NMR spectra were measured on Bruker AMX 500 spectrometer (^1^H, 500.14 MHz) in D_2_O:DMSO (3:2 *v*/*v*) mixture at RT. XPS measurements were carried out using a PHI 5000 Versa Probe II (ULVAC-PHI, Chigasaki, Japan) spectrometer with a monochromatic Al Kα radiation source (E = 1486.6 eV). A dual-beam charge compensation was used to avoid possible charging of samples. High resolution spectra were recorded with the analyzer pass energy set to 46.95 eV. All binding energies were corrected to C–C line at 284.8 eV. Deconvolution of obtained spectra was done with PHI MultiPak software. The spectrum background subtraction was done using the Shirley method.

### 2.6. Determination of the Nanohydrogel Particles Size and Morphology (DLS, SEM, TEM, AFM)

A Malvern Nano ZS light-scattering apparatus (Malvern Instrument Ltd., Worcestershire, UK) was used for dynamic light scattering (DLS) and zeta potential measurements. The Z-averaged hydrodynamic mean diameters (dz), polydispersity index (PDI), and distribution profiles of the samples were calculated using the software provided by Malvern. Samples were irradiated with the 633 nm laser beam at a fixed scattering angle of 173° at 25 °C. Every received value was an average of five tests and for each test an average of 3 measurements (each being a mean value of 10 repetitions) was determined. Surface morphology of the prepared NGs was studied using scanning electron microscopy (SEM), atomic force microscopy (AFM) and transmission electron microscopy (TEM).

SEM observations were performed with a Versa 3D FEG/SEM (FEI Company, Hillsboro, OR, USA) operating at an accelerating voltage of 10 kV using a secondary electron (SE) detector. The obtained NGs aqueous suspensions were deposited on silicon substrates using the spin coater and dried. The samples were coated with graphite using a Q150T E Plus sputter coater (Quorum Technologies, Laughton, UK) to make them conductive and mounted on a aluminum stub by double-faced carbon tape. Atomic force microscope (AFM) images were obtained with a Dimension Icon atomic force microscope (Bruker, Santa Barbara, CA, USA) working in the air in the PeakForce Tapping (PFT) mode using standard silicon cantilevers of nominal spring constant of 0.4 N/m. For both methods samples were prepared on previously prepared silica plates coated with poly(allylamine hydrochloride) (PAH) using a spin-coating technique. TEM measurements were performed using Tecnai TF 20 X-TWIN (FEI, Hillsboro, OR, USA). Nanohydrogel was deposited onto ultrathin carbon coated copper TEM grid. The TEM images were formed by STEM mode at an accelerating voltage of 200 kV.

### 2.7. UV/Vis Absorption Spectra Measurements

Binding constants (*K_a_*) of piroxicam molecules to polysaccharide derivatives (C-PUL and DEX-A) were determined by the UV-Vis measurements using a spectroscopic titration technique. Details of this technique have been described earlier [41]. The changes in absorbance of the solution containing piroxicam at the concentration [*P*] ranging from 3 × 10^−6^ to 7 × 10^−5^ M and polymer concentration [*H*]_0_ = 1 mg/mL were followed in water. Constant *K_a_* was calculated based on Benesi—Hildebrand Equation (1):

(1) $$\frac{1}{\Delta A}=\frac{1}{b\varepsilon^{PH}{\left[H\right]}_{0}K_{a}}\cdot \frac{1}{\left(\left[P\right]\right)}+\frac{1}{b\varepsilon^{PH}{\left[H\right]}_{0}}$$

where: Δ*A*—the difference between the absorbance *A* at any point of the binding process and the initial absorbance *A*_0_ in the absence of piroxicam ($\Delta A=A-A_{0}$); *ε*—the differential extinction coefficient; *l*—the optical path length.

UV/Vis absorption measurements were carried out in 1 cm quartz cuvette at 25 °C with Thermo Scientific Evolution 220 spectrophotometer (Thermo Fisher Scientific, Waltham, MA, USA) equipped with a Peltier temperature control (Thermo Fisher Scientific, Waltham, MA, USA). The samples were continuously stirred with a magnetic stirrer.

### 2.8. Determination of the Entrapment Efficiency of the Nanogel

Evaluation of the entrapment efficiency (*EE*%) of piroxicam inside NGs was done by pelletizing the sample at 16,000 rpm for 15 min at 4 °C. The obtained pellet was re-dispersed in water and then was lyophilized. Then, a 2 mg of sample (after lyophilization) was placed in 4 mL of methanol. The solution was sonicated using a sonic bath for 20 min. The obtained solution was centrifuged once more at 16,000 rpm for 15 min and the supernatant was collected. The concentration of the piroxicam in the supernatant was quantified spectrophotometrically at 358 nm, which corresponds to the maximum of piroxicam absorption band.

Entrapment efficiency (*EE*) was calculated based on Equation (2):

(2) $$EE\left(\%\right)=\frac{\mathrm{weight}\;\mathrm{of}\;\mathrm{piroxicam}\;\mathrm{in}\;1\;\mathrm{mg}\;\mathrm{of}\;\mathrm{the}\;\mathrm{pellet}}{\mathrm{initial}\;\mathrm{weight}\;\mathrm{of}\;\mathrm{piroxicam}\;\mathrm{added}\;\mathrm{per}\;1\;\mathrm{mg}\;\mathrm{of}\;\mathrm{the}\;\mathrm{pellet}}\;\times \;100$$

Loading efficiency (*LE*) was determined by applying the Equation (3):

(3) $$LE\left(\%\right)=\frac{\mathrm{weight}\;\mathrm{of}\;\mathrm{piroxicam}\;\mathrm{in}\;\mathrm{the}\;\mathrm{pellet}}{\mathrm{weight}\;\mathrm{ofthe}\;\mathrm{pellet}}\;\times \;100$$

### 2.9. Piroxicam Release from NG-PIX

Two series of seven 2 mL samples of NG-PIX in water, each containing 0.04 mg of piroxicam, were prepared. To each one 2 mL of oleic acid was added and all the samples were incubated in a proper temperature (37 °C) with constant agitation (50 rpm). After determined time periods, one sample of each series was taken out, the upper, oleic phase was separated and its absorbance at λ = 358 nm (the maximum of absorption band characteristic of piroxicam in oleic acid) was measured. The obtained absorbance value was then recalculated to obtain the amount of released piroxicam.

### 2.10. Biological Studies

Cell line NIH3T3 (murine fibroblasts) was cultured in medium DMEM (high glucose) in a humidified incubator under the following conditions: temperature, 37 °C; atmosphere of, 5% CO_2_. DMEM was supplemented with penicillin (100 μg/mL), streptomycin (100 U/mL) and 10% FBS. Every two days, the cells were subcultured until the appropriate number of cells to be tested was obtained. When the plate confluency was 80%, the cells were trypsinized and then inoculated into sterile 96-well plates (7 × 10^4^ cells/cm^2^) and incubated for 24 h. 50 mg/mL stock solution of polymer (C-PUL, PUL, DEX and DEX-A) prepared in PBS to diluted with culture media to make solutions of 0.1, 0.2, 0.3, 0.4, 0.5, 0.6, 0.7, 0.8, and 1.0 mg/mL. pH of all solutions was the same like in normal growth media. A 10 mM stock solution of piroxicam in DMSO was diluted with DMEM to achieve the desired concentration.

The cytotoxic activity of piroxicam in NIH3T3 line was estimated by the MTT assay [42,43]. 7 × 10^4^ fibroblasts were cultured in 100 μL volume of DMEM medium in 96-well plates in the presence of various concentrations of polymers (C-PUL, PUL, DEX, DEX-A), as well as in the presence of piroxicam dissolved in DMSO. Moreover, fibroblasts were cultured in the presence of the nanogel systems C-PUL/DEX-A type 4:1 with and without piroxicam. 24 h after stimulation, the cells were washed with PBS and further incubated with MTT dye. The obtained blue formazan crystals were dissolved in a solution of 5 mM HCl in isopropanol and incubated for 1 h at 37 °C. Absorbance measurements were made at a wavelength of 570 nm (with a correction of 630 nm) using a microplate reader SPECTROstar Nano from BMG LABTECH GmbH (Ortenberg, Germany). Each result shows the mean of three independent experiments, each carried out in triplicate standard. For each value, SD was also calculated, which is presented in charts, respectively. To compare the two groups, the Student’s t-test was used to determine the significance of differences between cell viability values. The probability value (*p*-value) in all cases when it was less than 0.05 was considered significant.

The preparations for confocal microscopy were obtained by fixing the cells with and without stimulation of nanogels containing piroxicam, in 1% paraformaldehyde in PBS for 20 min at 20 °C. An inverted Nikon Ti-E microscope with a Nikon A1 confocal system using a 405 nm diode laser for excitation was used for fluorescent imaging of the cells. Images were obtained in emission mode with a 60× objective. Fluorescence images of piroxicam which penetrated into the cells were measured using a 32-channel spectral detector. The emission image was recorded using a 458 nm barrier filter to remove cell-derived fluorescent background. The image size was 2048 × 2048.

### 2.11. Langmuir Trough Experiments

Isotherms of surface pressure-molecular area were measured using Langmuir-Blodgett trough-KSV NIMA, model large (Coventry, UK). Surface pressure was recorded (±0.1 mN/m) using a Wilhelmy plate made of filter paper linked to an electrobalance. Repeated cleaning of the surface subphase was performed until the absence of surface pressure changed between the “open” and “closed” positions. PCCP in chloroform solutions were spread to the water surface with a Hamilton microsyringe. All experiments were performed in triplicate.

## 3. Results and Discussion

### 3.1. Synthesis and Characterization of Cationic Pullulan Derivative

**Figure 1:**
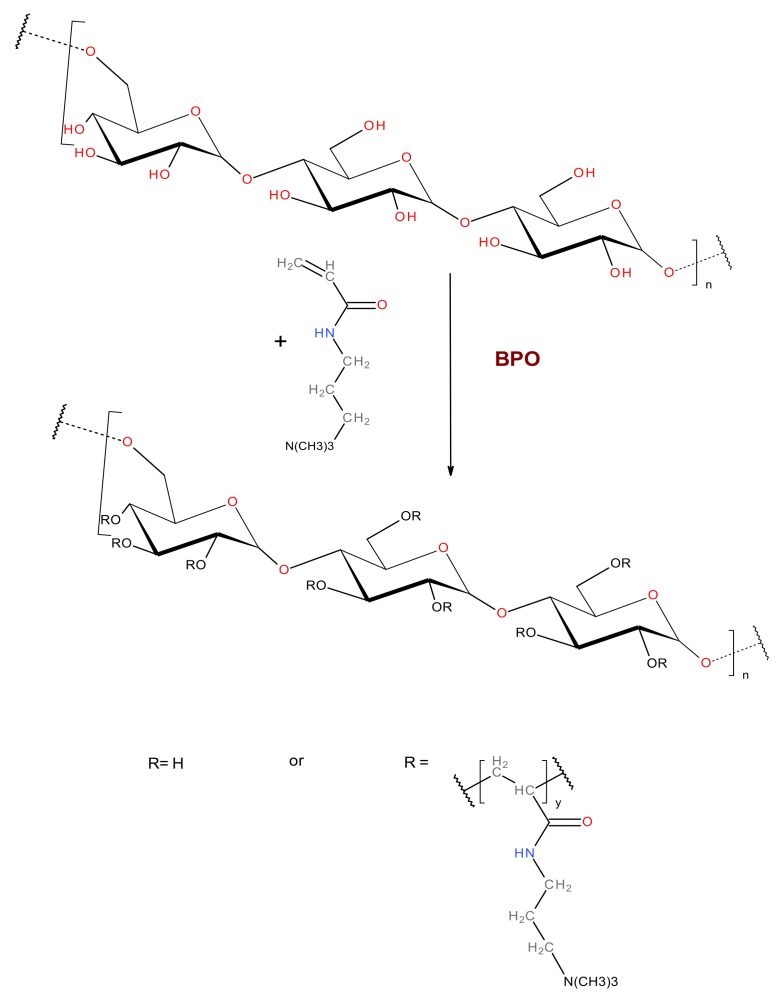
General synthetic scheme for cationic derivative of pullulan (C-PUL).

**Figure 2:**
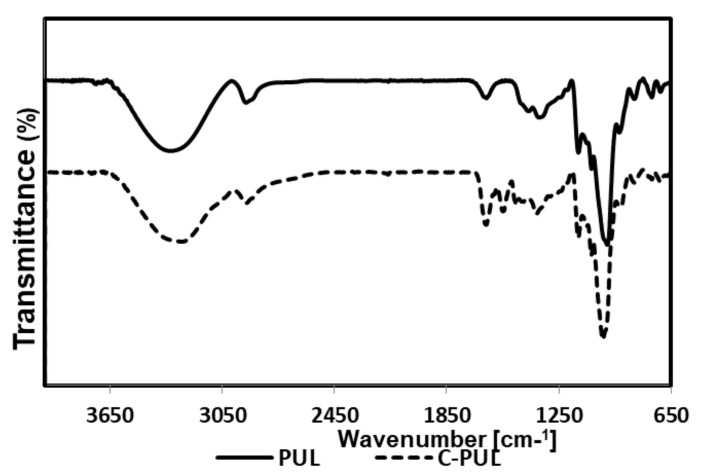
FT-IR spectra of pullulan (upper) and C-PUL (lower).

The cationic pullulan, C-PUL was obtained by grafting polymer chains containing trimethylammonium groups onto the main polysaccharide chain (Figure 1). The pullulan derivative was prepared in the reaction of pullulan and *N*-acrylamidopropyl-*N*,*N*,*N*-trimethylammonium chloride (APTMAC) in the presence of a radical initiator, BPO, in DMF at 70 °C. In order to confirm the successful modification of pullulan, FTIR spectra were measured for pullulan and its derivative C-PUL (Figure 2). FTIR spectrum of C-PUL shows a weak band at 1543 cm^−1^ which can be attributed to the asymmetric angular vibrations of the methyl groups of APTMAC. In the spectrum of the C-PUL bands at 1367, 1260, and 1000 cm^−1^ which correspond to the vibrations of hydroxyl groups have lower intensity than in the spectrum of unmodified pullulan, confirming further the successful derivatization.

**Figure 3:**
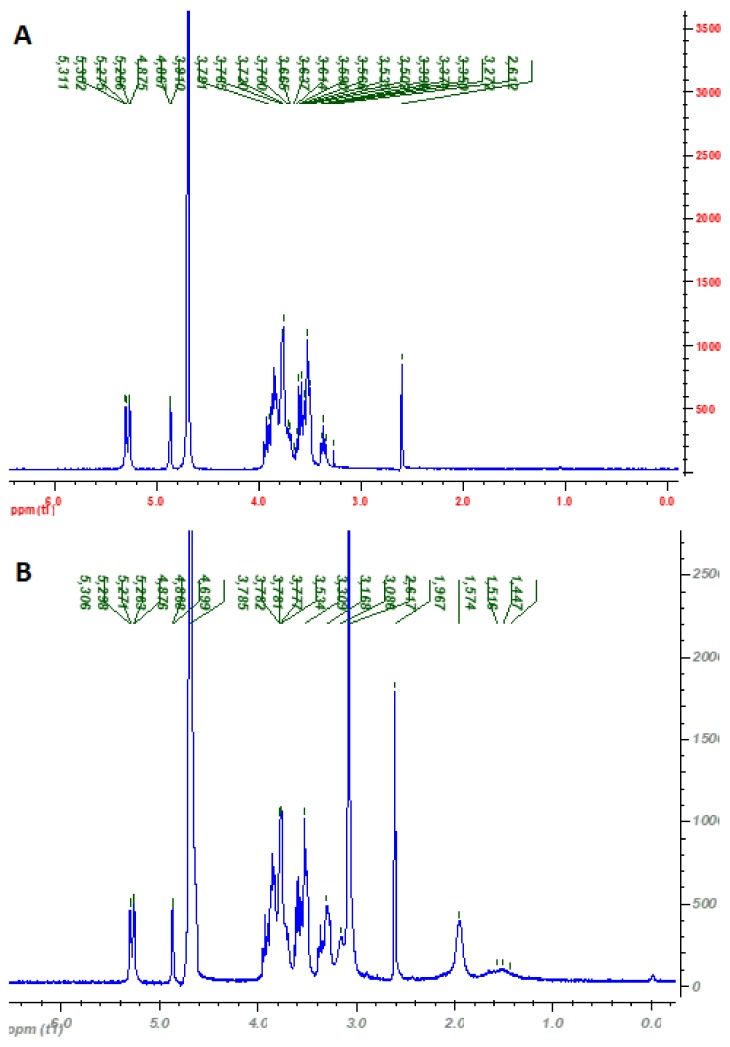
^1^H-NMR spectra of: (**A**) original pullulan (c = 2 mg/mL in D_2_O) measured at 25 °C; and (**B**) cationic pullulan (C-PUL) (c = 2 mg/mL in D_2_O) measured at 25 °C under the same instrumental conditions.

The structure of the cationic pullulan was also confirmed using ^1^H NMR (Figure 3). ^1^H NMR spectrum of C-PUL shows additional signals in the region of 1.4–1.5 ppm and 2 ppm, which can be assigned to the protons of the –CH and –CH_2_ groups resulting from the acrylic group of ATPMAC after binding to pullulan. Furthermore, a new, strong signal at 3.068 ppm was found which was ascribed to the nine protons of the –N^+^(CH_3_)_3_ group (Table S1).

**Figure 4:**
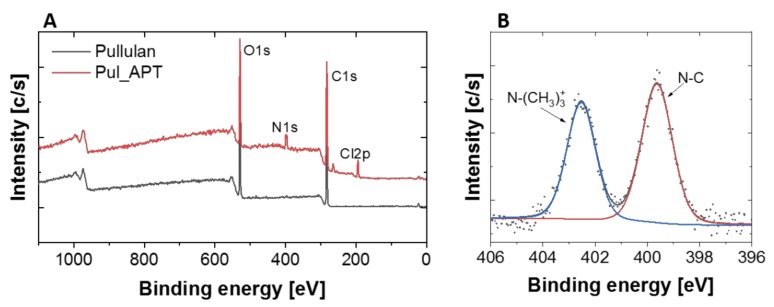
XPS spectra of: (**A**) pullulan and C-PUL survey; (**B**) N1s spectrum of C-PUL.

Additionally, the surface chemical composition of pullulan and C-PUL was studied with XPS wide energy range scan (Figure 4A). As expected, it confirmed the presence of carbon and oxygen in unmodified polymer with additional signals assigned to chloride and nitrogen in C-PUL. To study surface chemical bonds, high resolution spectra were made. N1s region consisted of two lines, −399.6 eV and 402.5 eV, which corresponded to N–C and N–(CH_3_)_3_^+^ bonds, respectively (Figure 4B). The presence of chloride (as counterion) was a result of synthesis path and was unavoidable. The value of degree of pullulan modification obtained from the XPS analysis was DS = 12.1 ± 0.91% (based on N/C ratio, Table S1).

### 3.2. Preparation of Nanohydrogel (NGs)

Nanohydrogel was formed after mixing solutions of the two polyions: cationic pullulan (C-PUL) and anionic dextran (DEX-A). To obtain a stable colloidal NGs suspension the series of different systems was prepared, each system differing in the ratio of polycation to polyanion (C-PUL/DEX-A) used. An average diameter of the NGs particles was determined for each system by DLS measurements (Table 1). For all the obtained nanogel systems the zeta (ζ) potential was measured (Table 1). All obtained hydrogel systems were characterized by short-term stability, which enabled stable and repeatable DLS measurements. Unfortunately, many nanogels did not exhibit long-term colloidal stability (low absolute values of ζ). To obtain a physically stable suspension, the absolute value of ζ potential should exceed ± 20 mV. Of the tested nanogel systems, only two had sufficiently large zeta potential: those obtained using the C-PUL: DEX-A ratio of 4:1 and 1:4.

**Figure 5:**
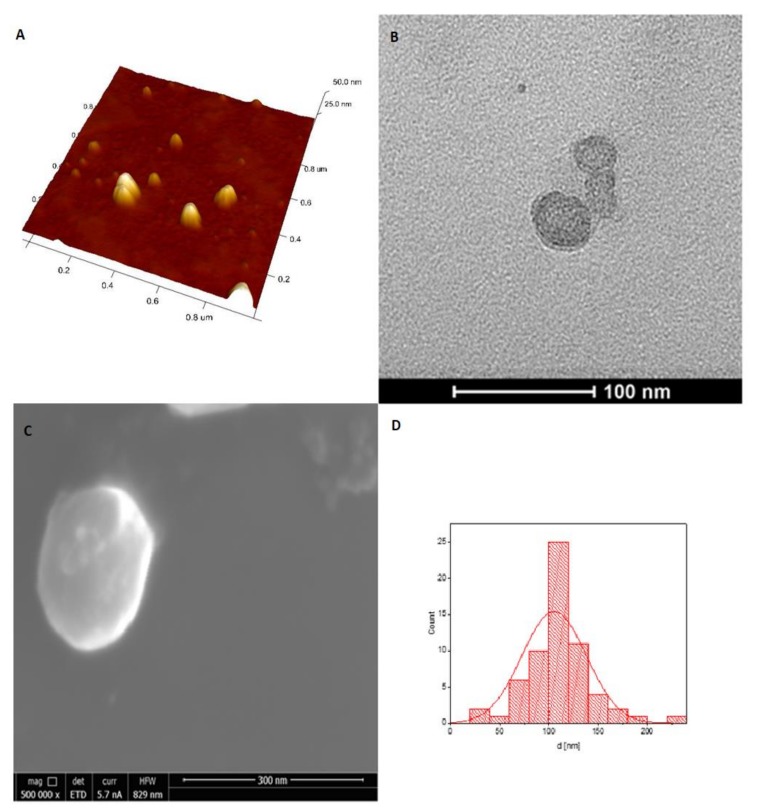
(**A**) Three dimensional (3D) AFM; (**B**) TEM; and (**C**) SEM images of C-PUL/DEX-A nanohydrogel structures (at C-PUL:DEX-A ratio of 4:1); (**D**) 4:1 NGs histogram obtained from SEM images (SI), fitted with a normal size distribution.

The obtained NGs particles were characterized by a spherical shape and a smooth surface, aspresented in the AFM (Figure 5A), TEM (Figure 5B) and SEM images (Figure 5C) for an example of 4:1 C-PUL/DEX-A system. The diameters of the NGs particles were in the range of 100–160 nm with exception of 1:4 NGs particles, which were larger. Due to its small size and low polydispersity index (PDI) the 4:1 C-PUL/DEX-A system was chosen for further studies. The morphology of this system was studied using various microscopic methods. The obtained nanogel system was characterized by a spherical shape and a smooth surface, as shown in the microscopic images (Figure 5). The average size of the nanogel particles, determined from all presented microscopy techniques (SEM, TEM, and AFM), was found to be in the range of 80–130 nm. The diameter obtained for the same system using the DLS technique was found to be equal to 127 ± 1 nm, which is in a very good agreement with all previous methods (Figure S1). This size is suitable for many drug delivery applications because it allows nanohydrogel particle to avoid uptake by the mononuclear phagocyte system. Therefore a 4:1 C-PUL/DEX-A system was chosen for further studies also due to its small size and low polydispersity index (PDI).

### 3.3. C-PUL-Binding Constant of Piroxicam

**Figure 6:**
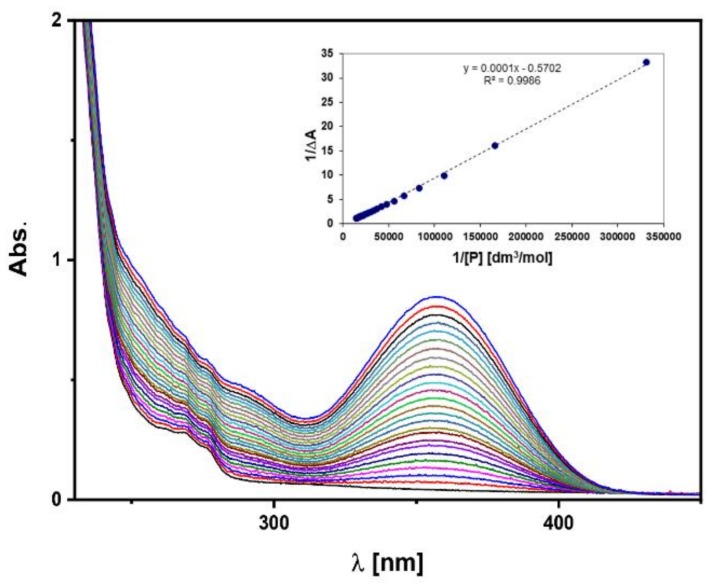
Absorption spectra of the C-PUL solutions in the presence of various concentrations of piroxicam (c_C-PUL_ = 1 mg/mL; piroxicam concentration range: 0–0.07 [mM]. The insert presents 1/Δ*A* as a function of 1/[*P*] and the linear fit.

The affinity of piroxicam to the cationic derivative of pullulan can be quantitatively described by a binding constant, *K_a_*. The value of *K_a_* to C-PUL was determined for piroxicam in water (pH = 7.0) using spectrophotometry. To study the one-to-one binding process between host and guest molecules using absorbance measurements, the Benesi—Hildebrand method is usually used [41,44]. Figure 6 shows a set of absorption spectra of the cationic pullulan solutions of C-PUL with different concentrations of piroxicam added. In the insert, a double reciprocal plot (1/Δ*A* versus 1/[*P*]) and the obtained fit are presented. *K_a_* was determined from this plot based on Equation (1) to be (3.6 ± 0.2) × 10^3^ M^−1^. This value is one order of magnitude smaller than the *K_a_* value determined for BSA/piroxicam system, which was reported in the literature [45]. But this value is sufficient to transport piroxicam by the proposed carrier.

### 3.4. Piroxicam-Loaded Nanohydrogel Particles (PIROX-NGs)

Piroxicam was entrapped inside the 4:1 C-PUL/DEX-A nanogel system during the self-assembly process of C-PUL and DEX-A. A methanolic solution of piroxicam was first added dropwise to the cationic pullulan solution. Methanol was then evaporated and the solution of anionic dextran DEX-A was added, while constantly stirring. Encapsulation efficiency (*EE*) and loading efficiency (*LE*) were determined for this system to be, respectively: *EE* = 57.8%, and *LE* = 1.6%.

### 3.5. Studies on the Intercalation of NGs Systems into the Lipid Monolayer

**Figure 7:**
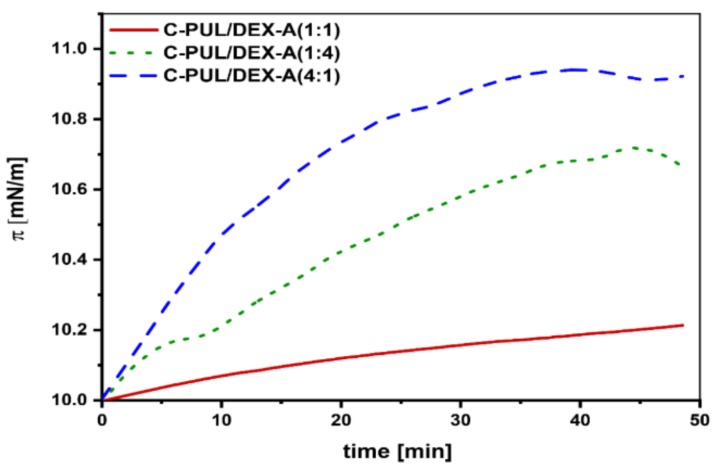
Changes in the surface pressure with time after spreading of 50 μL of a nanogel systems C-PUL/DEX-A at the different ratio on a EYPC monolayer compressed to 10 mN/m.

One of the crucial properties of a drug delivery system, besides high stability, is the ability to penetrate cell membranes. To this aim, a model interaction studies of the NGs carrier system with the lipid monolayer were performed using a Langmuir balance. Egg yolk phosphatidylcholine (EYPC) was used to form the model monolayer. First the lipid monolayer from EYPC was compressed to 10 mN/m. After an equilibration period of approx. 30 min, 50 μL of the appropriate piroxicam nanogel system solution were applied to the monolayer. Changes in the surface pressure (π) were then recorded for 50 min (Figure 7). For all tested C-PUL/DEX-A nanogel systems (4:1, 1:4, and 1:1 ratios), an increase in surface pressure was recorded, which can be associated with the intercalation of surface active nanogel particles to the EYPC monolayer. The largest effect was found in the case of 4:1 A-PUL/DEX-A system, confirming further the supremacy of this nanogel over other tested C-PUL/DEX-A systems.

### 3.6. Piroxicam Release

**Figure 8:**
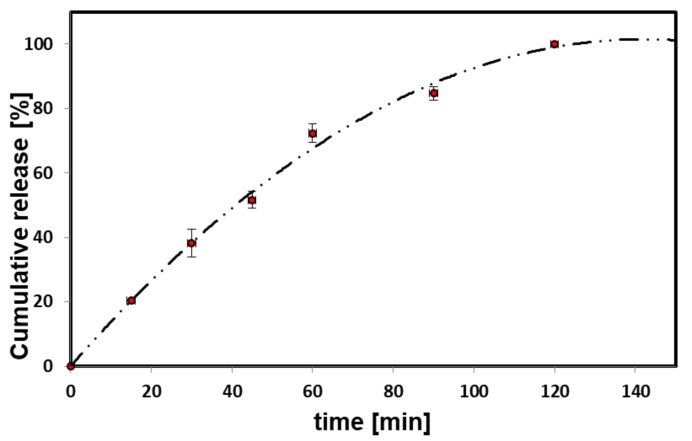
Piroxicam release profile (37 °C, pH = 7) from 4:1 C-PUL/DEX-A nanohydrogel. The error bars represent mean and standard deviations of the single experiments (*n* = 3).

To determine the suitability of the produced nanogel system as a carrier of piroxicam, the release process was also investigated. Preliminary physicochemical studies indicated 4:1. C-PUL/ DEX-A nanogel as the most promising, therefore the release studies were performed for this system. Piroxicam was gradually released from the nanogel particles (Figure 8, Figure S2). No “burst effect” was observed. The obtained release profile is almost linear up to 80 min and achieves plateau after 2 h. 

### 3.7. Biological Studies

**Figure 9:**
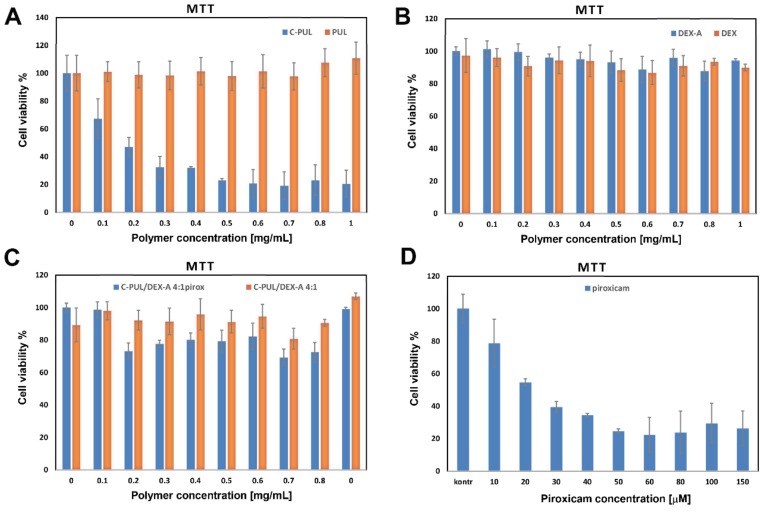
MTT assay test performed for NIH3T3 cells incubated for 24 h with (**A**) PUL and C-PUL; (**B**) DEX and DEX-A; and (**C**) 4:1 C-PUL/DEX-A nanogel systems without and with entrapped piroxicam (**D**) piroxicam in DMSO. Cell viability is expressed as a percentage of the control, which was defined as 100%. The error bars represent mean and standard deviations of the single experiments performed in triplicate (*n* = 3). DEX-A, anionic dextran sulfate.

To verify further the possible application of 4:1 C-PUL/DEX-A nanogel as piroxicam carrier, it was necessary to test their cytotoxicity, as well as cytotoxicity of the polymer components: C-PUL and the DEX-A polymers themselves. The viability of murine fibroblasts (NIH3T3 cell line) treated with C-PUL and unmodified pullulan was determined using MTT cell viability test (Figure 9). The results presented in the graph are the average value calculated from three parallel experiments (triplet standard). They indicate that while C-PUL is cytotoxic above 0.1 mg/mL, both DEX-A and C-PUL/DEX-A systems are safe even at 1 mg/mL. The cytotoxic effect of cationic pullulan, not observed for original pullulan, can be explained by the presence of the cationic group in the polymer structure. On the other hand, introduction of anionic group did not significantly increase toxicity of the resulting polymer, DEX-A. The increased cytotoxicity of cationic polymers was observed before [46,47] and may be associated with stronger electrostatic interactions between polycations and negatively charged cell membrane. Once C-PUL binds electrostatically to DEX-A its charge is shielded and cytotoxic effect is practically abolished. The presence of piroxicam in the nanogel slightly increases its cytotoxicity (decrease in viability by approximately 20%). The effect is, however, significantly weaker than for the free piroxicam (Figure 9D).

**Figure 10:**
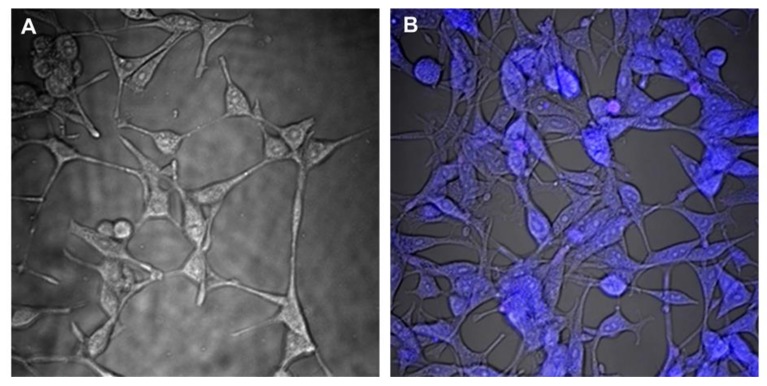
Confocal images for murine fibroblast NIH3T3 cell line (60× objective, 2× zoom) recorded at 24 h after stimulation with (**A**) “empty” 4:1 C-PUL/DEX-A nanogel (control); and (**B**) 4:1 C-PUL/DEX-A system with encapsulated piroxicam. Excitation 408 nm laser line; emission filter: 458 nm barrier filter.

The process of penetration of piroxicam encapsulated in 4:1 C-PUL/DEX-A nanohydrogel particles into murine fibroblasts (NIH3T3 line) was examined by microscopic observation using confocal microscopy. The study used the fact that piroxicam exhibits characteristic fluorescence in the visible region (460 nm emission; excitation with 408 nm laser line), therefore it was used as a fluorophore. Cell uptake and location of piroxicam in the cells 24 h after stimulation were studied (Figure 10).

In the images, intense blue emission of piroxicam was observed in cells stimulated with piroxicam-containing nanogel carriers (Figure 10B), which was not observed for the cells incubated with “empty” nanohydrogel system (Figure 10A). Additionally, piroxicam was observed solely inside the cells with no visible blue fluorescence in the extracellular spaces. Thus within 24 h piroxicam encapsulated within nanohydrogel system is efficiently and completely taken up by NIH3T3 cells, locating within the whole cellular structure.

## 4. Conclusions

New, cationic derivative of pullulan was synthetized by grafting trimethylammonium groups on the polysaccharide backbone. The success of the modification was confirmed by FTIR, ^1^H NMR, and XPS spectroscopies. The obtained polycation, C-PUL, interacts electrostatically with commercially available anionic dextran sulfate (DEX-A) instantly and spontaneously forming in aqueous solution stable hydrogel particles of nanometric size and high positive surface charge. The size and stability of the NG can be tuned by adjusting the polycation:polyanion ratio in solution. Based on the results obtained for a series of nanohydrogel, a 4:1 C-PUL/DEX-A system was selected as optimal for potential drug delivery applications. A model drug, piroxicam, was shown to form complex with C-PUL, and its binding constant to C-PUL was determined to be (3.6 ± 0.2) × 10^3^ M^−1^. After complexation with pullulan derivative, piroxicam was successfully encapsulated in the 4:1 NG described above. The obtained drug-NG system was then shown to effectively intercalate to the lipid EYPC monolayer. Piroxicam release from the nanohydrogel was also studied. No “burst” release was observed; piroxicam was released gradually for 2 h with a profile close to linear within the first 80 min. Finally, the preliminary biological studies were performed, which showed that the obtained piroxicam-NG system had considerably lower cytotoxicity than a free drug, and that it was effectively taken up by cells within 24 h. We believe that this new nanohydrogel delivery system has great potential as a versatile, biocompatible system for safe and effective delivery of hydrophobic drugs.

## Figures and Tables

**Table 1:** Mean hydrodynamic diameter (d_Z_), polydispersity index (PDI) and ζ potential of the NGs systems obtained at different polycation/polyanion ratios.

C-PUL:DEX-A (*w*/*w*)	d_z_ (nm)	PDI	ζ (mV)
1:1	108 ± 2	0.359	−28.6 ± 3.3
1:2	106 ± 1	0.351	−22.4 ± 2.3
1:4	337 ± 2	0.298	−21.0 ± 2.0
1:6	165 ± 6	0.432	−38.4 ± 3.9
1:10	130 ± 8	0.733	−42.3 ± 4.3
2:1	156 ± 1	0.297	−16.2 ± 3.4
3:1	117 ± 2	0.195	+11.1 ± 1.0
4:1	127 ± 1	0.228	+19.7 ± 0.7
6:1	152 ± 10	0.445	+35.2 ± 1.4

## Supplementary Materials

The following are available online at https://www.mdpi.com/1999-4923/11/12/622/s1, Figure S1. DLS estimation of nanogel particles C-PUL/DEX-A (4:1 system with piroxicam) hydrodynamic 12 diameter in pH = 4.0 (buffer acetate). Figure S2. Piroxicam release profile (37 °C, pH = 4) from 4:1 C-PUL/DEX-A nanohydrogel. The error bars represent mean and standard deviations of the single experiments (*n* = 3). Table S1: Substitution degrees for the obtained cationic derivative of pullulan (C-PUL).
