# Supplementary material for: Nanohydrogels Based on Self-Assembly of Cationic Pullulan and Anionic Dextran Derivatives for Efficient Delivery of Piroxicam
Source: Pharmaceutics. 2019 Nov 21;11(12):622. doi: 10.3390/pharmaceutics11120622 (PMC6956171; doi:10.3390/pharmaceutics11120622)
Supplement: Supplementary file 1 [file pharmaceutics-11-00622-s001.pdf]

pharmaceutics

MDPI

# Supplementary Materials: Nanohydrogels Based on Self-Assembly of Cationic Pullulan and Anionic Dextran Derivatives for Efficient Delivery of Piroxicam

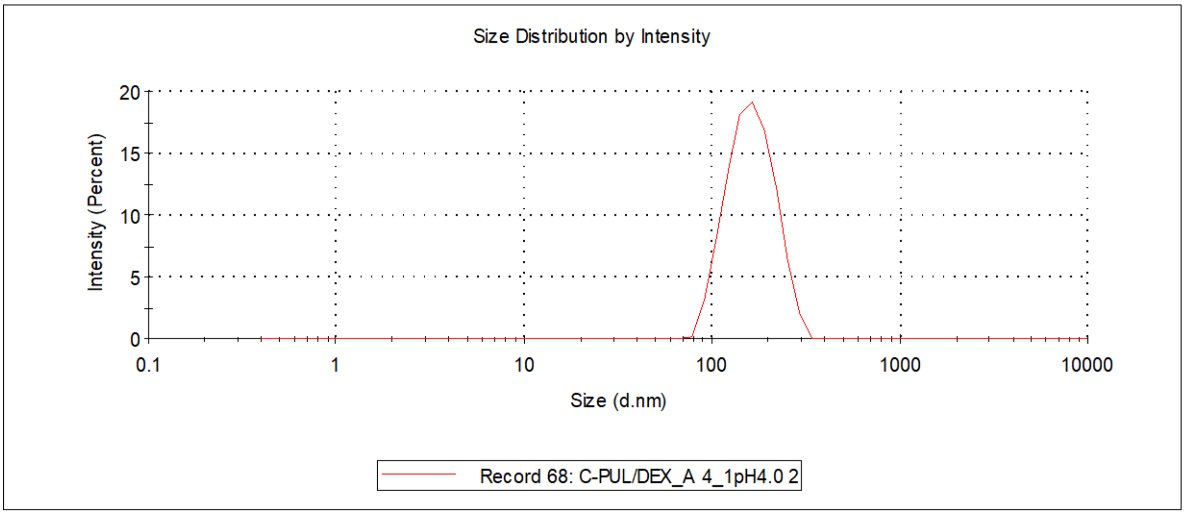


**Figure S1.** DLS estimation of nanogel particles C-PUL/DEX-A (4:1 system with piroxicam) hydrodynamic diameter in pH = 4.0 (buffer acetate).

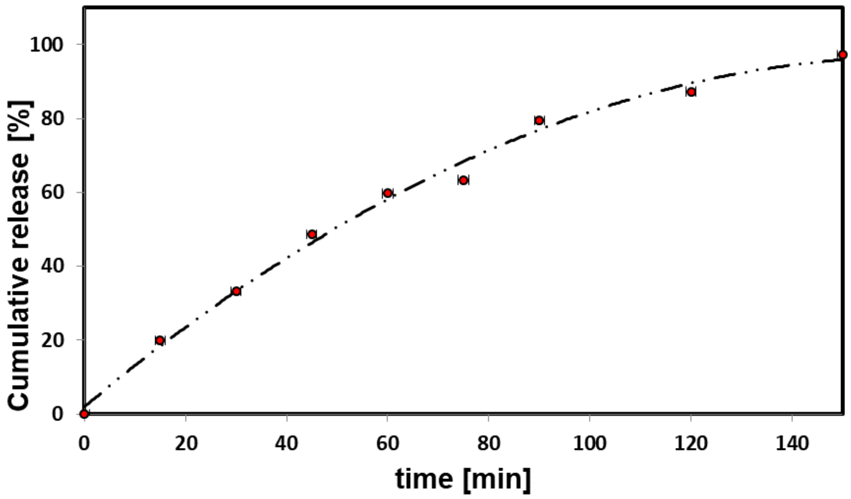


**Figure S2.** Piroxicam release profile (37 °C, pH = 4) from 4:1 C-PUL/DEX-A nanohydrogel. The error bars represent mean and standard deviations of the single experiments ($n$ = 3).

**Table S1.** Substitution degrees for the obtained cationic derivative of pullulan (C-PUL).

| Material | Degree of substitution (DS %) (based on $^1$H NMR spectrum) | Degree of substitution (DS %) (based on XPS spectrum) |
|---|---|---|
| C-PUL | 10.8 ± 2.21 | 12.1±0.91 |
